# Associations of physical activity in detailed intensity ranges with body composition and physical function. a cross-sectional study among sedentary older adults

**DOI:** 10.1186/s11556-020-0237-y

**Published:** 2020-01-24

**Authors:** Tiina Savikangas, Anna Tirkkonen, Markku Alen, Taina Rantanen, Roger A. Fielding, Timo Rantalainen, Sarianna Sipilä

**Affiliations:** 1 Gerontology Research Center and Faculty of Sport and Health Sciences, University of Jyväskylä, PO Box 35 (viv256), FIN-40014 Jyväskylä, Finland; 2 Department of Medical Rehabilitation, Oulu University Hospital, Oulu, Finland; 3 Nutrition, Exercise Physiology, and Sarcopenia Laboratory, Jean Mayer USDA Human Nutrition Research Center on Aging, Tufts University, Boston, MA USA

**Keywords:** Accelerometer, Physical performance, Walking speed, Fat percent, Community-dwelling

## Abstract

**Background:**

Physical activity is crucial to maintain older adults’ health and functioning, but the health benefits of particular activity intensities remain unclear. The aim of this cross-sectional study was to peruse the distribution of physical activity, and to investigate the associations of particular physical activity intensities with body composition and physical function among older adults.

**Methods:**

The sample comprised of 293 community-dwelling sedentary or at most moderately active older adults (42% men, mean age 74 ± 4 years). Physical activity was measured with a hip-worn tri-axial accelerometer over seven consecutive days, and investigated in detailed intensity range and in categories of sedentary, light and moderate-to-vigorous activity. Fat percent and appendicular lean mass were measured with DXA. Physical function was assessed by six-minutes walking test (6-min walk), maximal walking speed over 10 m (10-m walk) and Short Physical Performance Battery (SPPB). Associations were estimated with partial correlation adjusted for sex and age.

**Results:**

Participants spent on average 602 min per day sedentary, 210 min in light activity and 32 min in moderate-to-vigorous activity. Light and moderate-to-vigorous activity were negatively associated with fat percent (*r* = − 0.360 and *r* = − 0.384, respectively, *p* < 0.001 for both), and positively with SPPB, 10-m walk and 6-min walk results (*r* = 0.145–0.279, *p* < 0.01, for light and *r* = 0.220–0.465, *p* < 0.001, for moderate-to-vigorous activity). In detailed investigation of the intensity range, associations of physical activity with fat percent, 6-min walk and 10-m walk were statistically significant from very light intensity activity onward, whereas significant associations between physical activity and SPPB were observed mostly at higher end of the intensity range. Sedentary time was positively associated with fat percent (*r* = 0.251, *p* < 0.001) and negatively with 6-min walk (*r* = − 0.170, *p* < 0.01).

**Conclusion:**

Perusing the physical activity intensity range revealed that, among community-dwelling sedentary or at most moderately active older adults, physical activity of any intensity was positively associated with lower fat percent and higher walking speed over long and short distances. These findings provide additional evidence of the importance of encouraging older adults to engage in physical activity of any intensity. More intervention studies are required to confirm the health benefits of light-intensity activity.

## Background

Promoting physical activity and health of older adults is crucial. Deterioration of physiological functions and body composition together with declines in physical activity by aging are associated with deterioration of physical function [1] and loss of mobility [2]. Physical activity is known to counteract many of the unfavorable age-related changes in health and functioning [3]. For example, physical activity contributes to maintenance of healthy weight, cardiovascular health, muscular strength and physical functioning [3, 4]. In contrast, sedentary behavior has emerged as an independent risk factor for poor health and mortality [5], and has been associated with e.g., obesity [5], muscle weakness [6] and mobility disability [7] among older adults.

The health benefits of moderate-to-vigorous intensity activity for older adults are well-known [1, 4]. Participation in regular exercising maintains physical function [8]. Recent cross-sectional studies have consistently shown a positive association between habitual accelerometer-measured moderate-to-vigorous-intensity activity and better performance in physical function tests including endurance, strength and agility [9–14]. Higher levels of overall accelerometer-based physical activity and moderate-to-vigorous-intensity activity in particular may also help to maintain muscle mass in old age [15], but this is not supported by all studies [14]. A growing body of evidence indicates that even light-intensity activity may lower mortality risk [16, 17] and the risk of obesity [16], delay brain aging [18], and provide other health benefits for older adults [16]. Preliminary evidence from a cross-sectional study indicates that habitual accelerometer-based light-intensity activity may be beneficially associated also with physical function among older adults [10], but the data are still few and inconsistent. Other recent studies have shown no association between light physical activity and physical function [9, 11], or the association has not been significant throughout the spectrum of light-intensity activity or in both sexes [13]. Even though physical activity is known to maintain healthy weight, muscle strength and physical functioning in older age [3, 4], the associations of particular physical activity intensities with physical function and body composition remain unclear among older adults.

Despite the benefits of physically active lifestyle, many older people spend most of their awake time sedentary [19] and have difficulties to achieve or maintain moderate-to-vigorous-intensity activities in longer bouts [20]. In contrast, older adults often engage in lighter-intensity activities, such as casual walking or household activities [20]. For many sedentary older adults these activities may be significantly more strenuous than for young and fit individuals [4, 21], and the standardly defined cut-points for accelerometer-based moderate-intensity activity may thus underestimate the intensity of habitual physical activity among older adults [22]. Perusing accelerometer data in more detailed than in simple metrics, such as mean daily minutes in intensity categories or step counts, is therefore essential to widen our understanding of what physical activity metrics are significant for older adults’ health and functioning [23].

The purpose of this cross-sectional study was to describe the distribution of accelerometer-measured habitual daily physical activity in detailed intensity range utilizing a novel analysis approach, and in categories of sedentary, light and moderate-to-vigorous-intensity activity, and to investigate what intensities were associated with measures of body composition and physical function in a representative sample of community-dwelling, sedentary or at most moderately active 70–85 year old men and women.

## Materials and methods

### Study design and participants

**Fig. 1:**
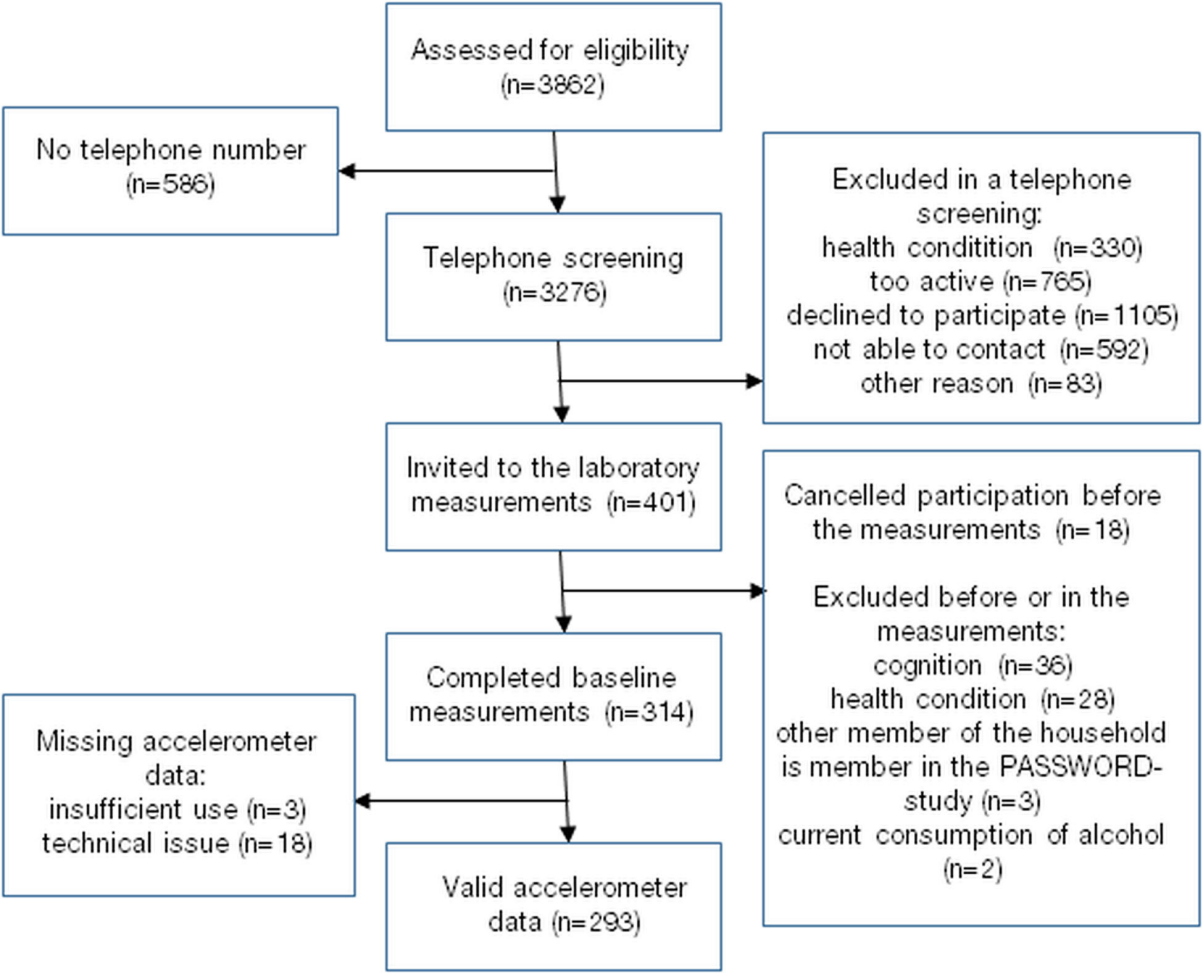
Flow chart of the study

This cross-sectional study utilized the baseline data of the PASSWORD -study. Recruitment process and measurements have been described in detail by Sipilä et al. [24]. To be included, participants had to be 70–85 year old, community-dwelling, able to walk 500 m without assistance, to be sedentary or at most moderately active (less than 150 min of walking/week and no attendance in resistance training) and to score ≥ 24 points in Mini Mental State Examination test (MMSE). Exclusion criteria were: severe chronic condition or medication; other medical, psychiatric, or behavioral factor that may interfere with study participation; excessive alcohol use; severe vision or hearing problem; other family member participating in the XX -study. In total, 314 men and women were recruited of which 293 had acceptable accelerometer data. Flow chart is shown in Fig. 1.

### Measurements

#### Physical activity

Tri-axial accelerometer, model UKK RM42 (UKK, Tampere, Finland) was used. Participants were instructed to wear the accelerometer seven consecutive days in an elastic waistband above the iliac crest on the right side during waking hours, except during water-based activities. Participants kept a diary of wearing hours as well as times and reasons for taking off the accelerometer for more than 30 min. Days with at least 10 h of wear-time were considered acceptable and data from participants with at least 3 acceptable days were included in the present report.

The UKK RM42-accelerometer measures and stores acceleration at 100 Hz sampling rate with 13-bit A/D conversion of the ±16 g range. Activity and inactivity thresholds of the devices were adjusted to account for the slower pace of movement of older adults. The recorded raw acceleration data were analyzed off-line with a custom-written MATLAB (version R2016b, The MathWorks Inc., Natick MA, USA) script. The Euclidian norm (resultant) of each acceleration sample was calculated, and further analysis was based on the resultant acceleration. The resultant was analyzed in five-second (5-s) non-overlapping epochs for mean amplitude deviation (MAD) [25]. That is, the mean of a given 5-s epoch was calculated and subtracted from the resultant accelerations, the absolute (negative signs were changed to positive) was taken from each value, and the mean of the absolute values was used as the 5-s MAD for the epoch. The epochs were divided into 24-h segments based on the diaries, and the mean of non-overlapping 1-min 5-s MAD epochs was calculated from mid-night to mid-night. Non-wear time was subsequently taken off as any epoch of at least 60-min with the 1-min MAD values continuously below 0.02 g. The non-wear algorithm resulted in good correspondence to the participant-reported diary-based wear-time.

The mean daily amount of physical activity was divided into two histograms based on the 1-min epochs. The first was based on the de facto standard of dividing the day into sedentary (bin threshold < 0.0167 g), light (≥ 0.0167 to < 0.091 g), moderate (≥ 0.091 to < 0.414 g), and vigorous (≥ 0.414 g) activities. Due to the very limited amount of vigorous-intensity activity, moderate and vigorous intensity activities were combined. The cut-points have been defined and validated against VO_2_ [25, 26], and compared with widely used Freedson’s cut-points for activity counts from uniaxial ActiGraph GT3X [27] in healthy younger adults, but not in older adults. In the last-mentioned study, MAD values showed slightly more sedentary activity, but notably less light activity and more moderate activity than activity counts. The amount of vigorous activity was similar [27]. To investigate the physical activity intensity range in detail, a second histogram with histogram bins from zero to 1.2 g in base 10 logarithmically equidistant bins was calculated [28], which resulted in 93 bins with at least some activity recorded. The use of logarithmically equidistant bins allows for a more detailed investigation of lower intensity activities, i.e. the bins are narrower at the lower intensities and wider at the higher intensities.

#### Body composition

Dual-energy x-ray absorptiometry (DXA, LUNAR Prodigy, GE Healthcare) was used to measure fat percent and appendicular lean mass. Participants were scanned in supine position in the center of the table using the default-scanning mode for total body automatically selected by the Prodigy software (Lunar Prodigy Advance Encore v. 14.10.022).

#### Physical function

Physical function measures included six-minutes walking distance (6-min walk) [29], maximal walking speed (m/s) over 10 m (10-m walk) [30] and Short Physical Performance Battery (SPPB) [31]. In 6-min walk participants walked a 20-m track back and forth in a comfortable pace without resting for 6 min, and total distance walked was recorded in meters. In 10-m walk, participants were asked to walk over the 10 m course as fast as possible without compromising safety. The fastest time of two trials was accepted as the result, and maximal walking speed was calculated (m/s). The SPPB assesses lower extremity functioning and includes habitual walking speed over four meters, five-time chair rise time and standing balance tests. The score varies between 0 and 12 and the higher score indicates better performance [31].

#### Background characteristics

Sex and date of birth were drawn from the population register. Anthropometrics were measured using standard procedures. Other background characteristics were drawn from a comprehensive questionnaire, and included highest education (low, i.e. primary school or less, medium, i.e. middle school, folk high school, vocational school or secondary school, vs. high, i.e. high school diploma or university degree), current self-perceived health (very good/good vs. average/poor), and current self-perceived mobility (very good/good vs. poor/very poor).

### Statistical analyses

Descriptive data are expressed as means and standard deviations (SD) for continuous variables and frequency (n) and percentage (%) for categorical variables in all participants and for men and women separately. To illustrate the distribution of physical activity along the whole intensity range, the mean minutes per day and number of participants having any recorded activity at each of the logarithmically equidistant intervals are presented as diagrams.

The associations of the mean daily minutes in sedentary, light and moderate-to-vigorous-intensity activity, with the body composition and physical function measures were assessed with partial correlation (Pearson) adjusted for sex and age. The associations of light-intensity activity with body composition and physical function indicators were further controlled for time spent in moderate-to-vigorous-intensity activity and vice versa. To investigate the strength of the associations along the whole physical activity intensity range, partial correlation coefficients were calculated for time spent at each of the logarithmically equidistant intervals and each body composition and physical function variable. Results are presented in graphs as correlation coefficient r and 95% confidence interval (CI). Graphs present correlations for activity intensities from 0.00188 g to 0.31 g since the first bin included the non-wear time and the amount of data on intensities exceeding 0.31 g was very limited. Statistical analyses were performed with IBM SPSS Statistics 24 (SPSS Inc., Armonk, NY). Statistical significance level was set at 0.05 for all analyses.

## Results

### Participant characteristics

Descriptive data are presented in Table 1. Mean age was 74 years, and 28 participants were ≥ 80. The average fat percent was 19% and participants had on average 36 kg of appendicular lean mass. In 6-min walk the mean distance completed was 478 m. The mean score in SPPB was 10 and the average speed in 10-m walk was 2 m/s.

**Table 1:** Descriptive statistics in full sample and according to sex (mean ± standard deviation or frequency (%))

	All (*n* = 293)	Men (*n* = 122)	Women (*n* = 171)
Age, years	74.44 ± 3.78	74.35 ± 3.90	74.50 ± 3.69
Anthropometrics			
Height, m	1.66 ± 0.09	1.73 ± 0.06	1.61 ± 0.06
Weight, kg	76.84 ± 14.35	84.07 ± 12.45	71.68 ± 13.39
Body mass index, kg/m^2^	27.88 ± 4.77	27.88 ± 3.60	27.87 ± 5.46
Waist circumference, cm	95.69 ± 12.47	102.11 ± 9.73	91.11 ± 12.20
Basic education, n (%)			
Low	43 (15)	25 (21)	18 (11)
Medium	186 (64)	77 (63)	109 (64)
High	64 (22)	20 (16)	44 (26)
Current self-rated health, n (%)			
very good/good	135 (46)	55 (45)	80 (47)
average/poor	158 (54)	67 (55)	91 (53)
Current self-rated mobility, n (%)			
very good/good	269 (92)	113 (93)	155 (91)
poor/very poor	25 (9)	9 (7)	16 (9)
Body composition^a^			
Fat percent	35.94 ± 8.23^a^	30.15 ± 6.01^a^	40.04 ± 7.04
Appendicular lean mass, kg	19.40 ± 4.37^a^	23.69 ± 2.95^a^	16.36 ± 2.05
Physical function			
6-min walk, m	477.55 ± 82.56	502.60 ± 90.68	459.68 ± 71.30
10-m walk, m/s	1.98 ± 0.38	2.11 ± 0.45	1.88 ± 0.29
SPPB, total score	10.19 ± 1.54	10.64 ± 1.45	9.87 ± 1.53
Accelerometer-measured physical activity			
Valid days	6.7 ± 0.8	6.7 ± 0.7	6.6 ± 0.8
Wear time, h/d	14.1 ± 1.3	14.3 ± 1.3	13.9 ± 1.2
Sedentary activity, min/d	602.3 ± 82.9	627.1 ± 81.0	584.6 ± 79.9
Light activity, min/d	210.3 ± 66.3	196.9 ± 60.8	219.8 ± 68.6
Moderate-to-vigorous activity, min/d	32.5 ± 20.1	33.1 ± 21.0	32.1 ± 19.5

Note

Abbreviations: 6-min walk = distance walked in 6 mins; 10-m walk = maximal walking speed over 10 m; *SPPB* Short Physical Performance Battery

^a^Missing *n* = 1

**Fig. 2:**
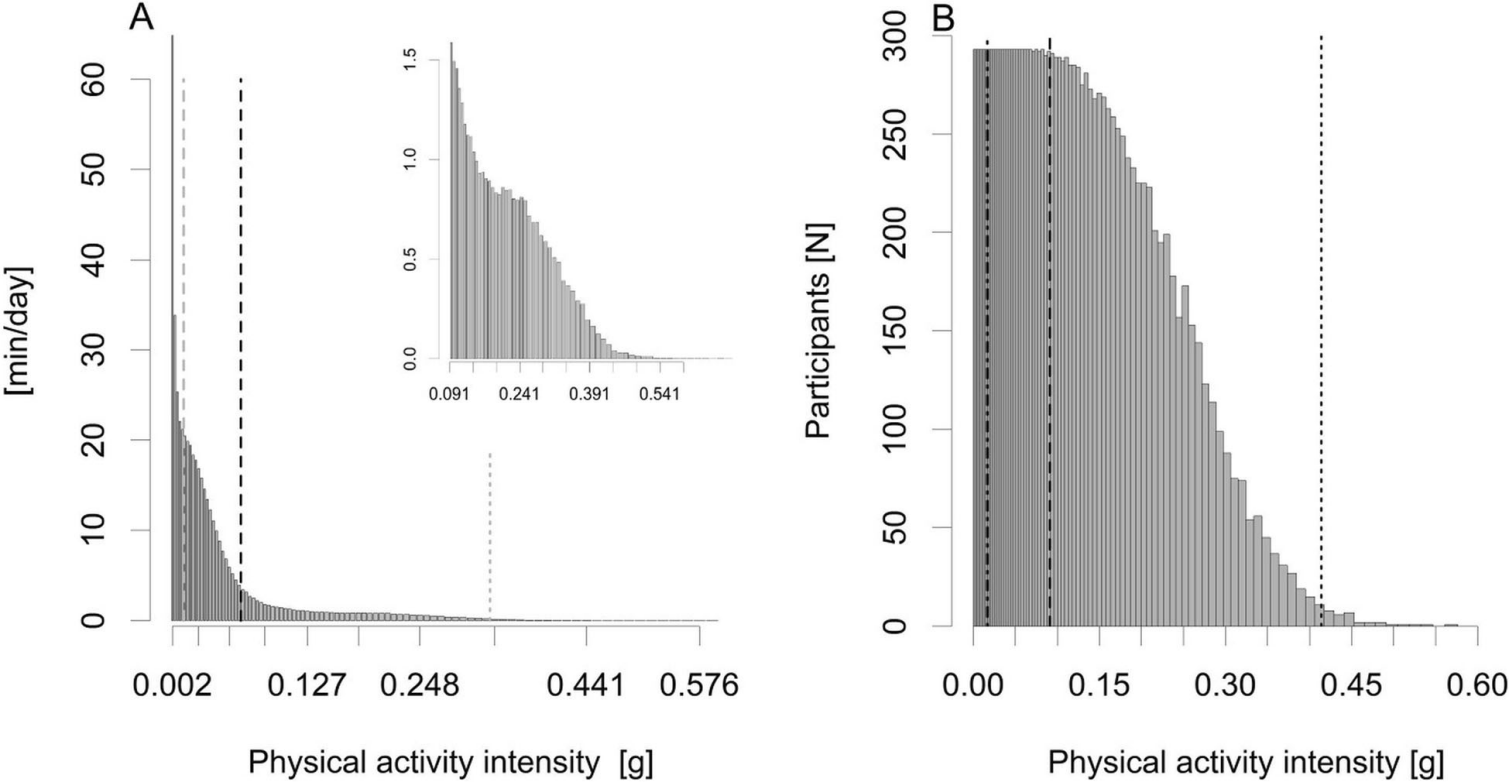
Distribution of physical activity in detailed intensity range. **a** for mean daily minutes (y-axis) at each of the logarithmically equidistant intervals along the whole intensity range (x-axis) from sedentary to vigorous intensity activity (0.00188 g to 0.62305 g), and within moderate-to-vigorous-intensity activity range (**a**, imputed small figure). **b** for number of participants (y-axis) having some activity at each interval (x-axis). The verticals mark the cut-points of light (0.0167 g), moderate (0.091 g) and vigorous-intensity activity (0.414 g)

Participants wore the accelerometer on average 14 h per day and had on average 6.7 acceptable measurement days. Participants spent on average 602 min, i.e. 10 hours, per day sedentary. Light-intensity activity covered on average 210 min (3.5 h) and moderate-to-vigorous-intensity activity 32 min (0.5 h) of mean daily wear-time (Table 1). Most of the active time was spent in very light-intensity activity with a drastic decrease from 19.4 min in the first to 1.7 min in the last bin within the light-intensity range (Fig. 2a). Within the moderate-intensity range, most time was spent at the lower intensities, the mean time spent at each of the intervals decreased gradually, and the amount of vigorous-intensity activity (≥ 0.414 g) was nearly non-existing. All participants had at least some moderate-intensity activity (≥ 0.091 to < 0.414 g) (Fig. 2b). A steep decline was observed in the number of participants having some activity exceeding 0.16 g. Less than one third of participants reached accelerations exceeding 0.31 g, and only few had any vigorous-intensity activity.

### Associations of accelerometer-measured physical activity with body composition and physical function

Time spent in sedentary activity was positively associated with fat percent and negatively associated with 6-min walk (Table 2). Time spent in both light and moderate-to-vigorous-intensity activities was negatively associated with fat percent and positively associated with 6-min walk, 10-m walk and SPPB. Appendicular lean mass was not associated with any physical activity intensity category (Table 2). Adjusting the associations of light activity with body composition and physical function for time spent in moderate-to-vigorous activity and vice versa did not notably change the results except that the association between light activity and SPPB was no longer significant.

**Table 2:** Partial correlations of physical activity in intensity categories with body composition and physical function

	Sedentary activity	Light activity		Moderate-to-vigorous activity	
	Model 1	Model 1	Model 2	Model 1	Model 3
Fat percent^a^	0.251***	−0.360***	−0.281***	−0.384***	− 0.312***
Appendicular lean mass^a^	0.006	−0.014	−0.018	0.010	0.015
6-min walk	−0.170**	0.279***	0.168**	0.465***	0.418***
10-m walk	−0.101	0.203**	0.122*	0.315***	0.273***
SPPB	−0.028	0.145**	0.086	0.220***	0.188**

Note

Abbreviations: 6-min walk = distance walked in 6 mins; 10-m walk = maximal walking speed over 10 m; *SPPB* Short Physical Performance Battery

Model 1: Controlled for sex and age

Model 2: Controlled for sex, age and moderate-to-vigorous activity

Model 3: Controlled for sex, age, and light activity

^a^Missing *n* = 1

*p < 0.05, ** *p* < 0.01, *** *p* < 0.001

**Fig. 3:**
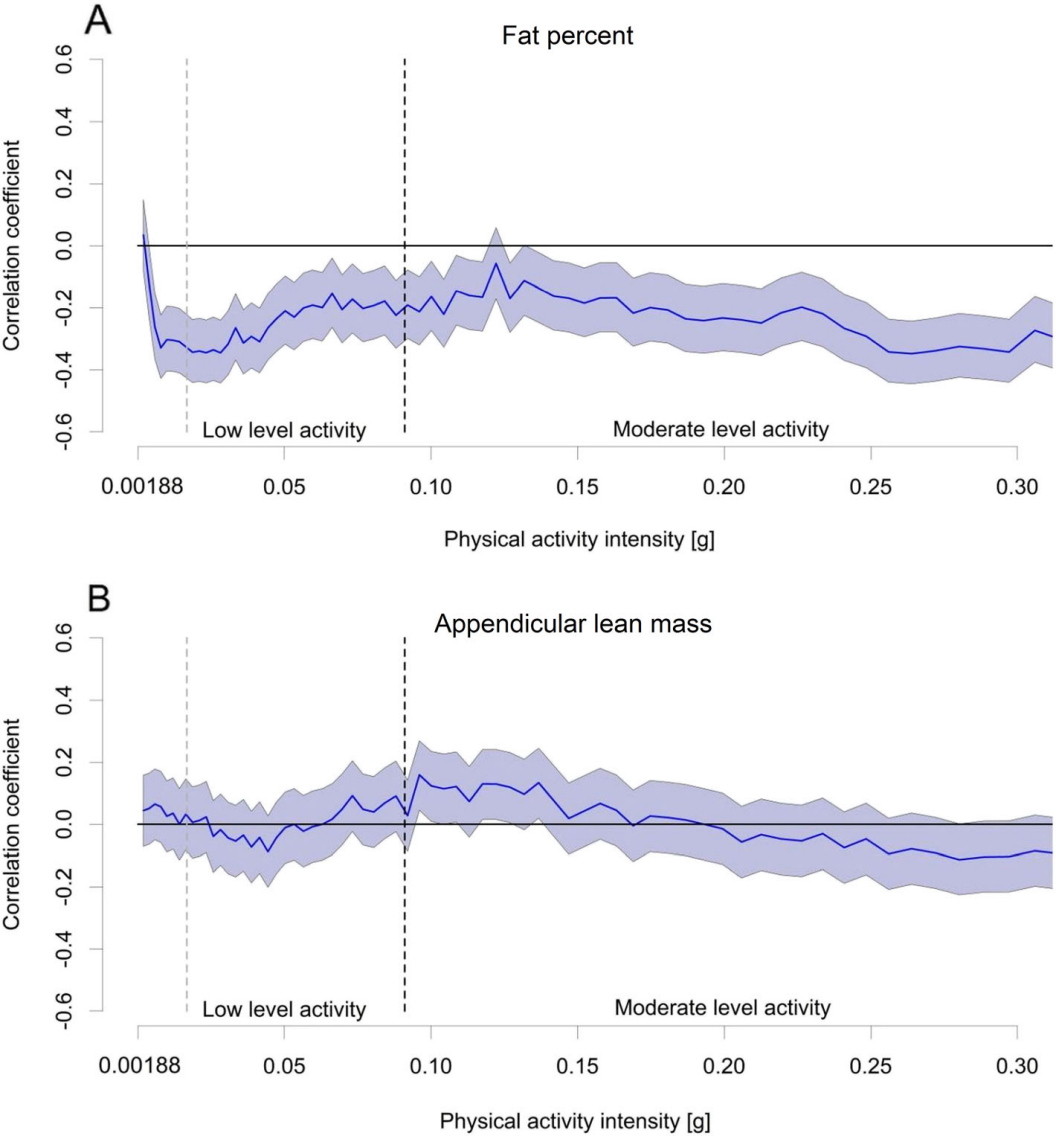
Associations of physical activity intensities from 0.00188 to 0.31 g with body composition. Associations of mean daily minutes at each physical activity intensity bin with fat percent (**a**) and appendicular lean mass (**b**) are expressed as mean correlation coefficient r (y-axis, black line) and 95% confidence interval (CI, shaded area). Physical activity intensities are shown in the x-axis. Associations are statistically significant, if the 95% CI area does not cross the 0-line. Verticals mark the cut-points for light-intensity activity (0.0167 g) and moderate-intensity activity (0.091 g). Correlations are adjusted by sex and age

**Fig. 4:**
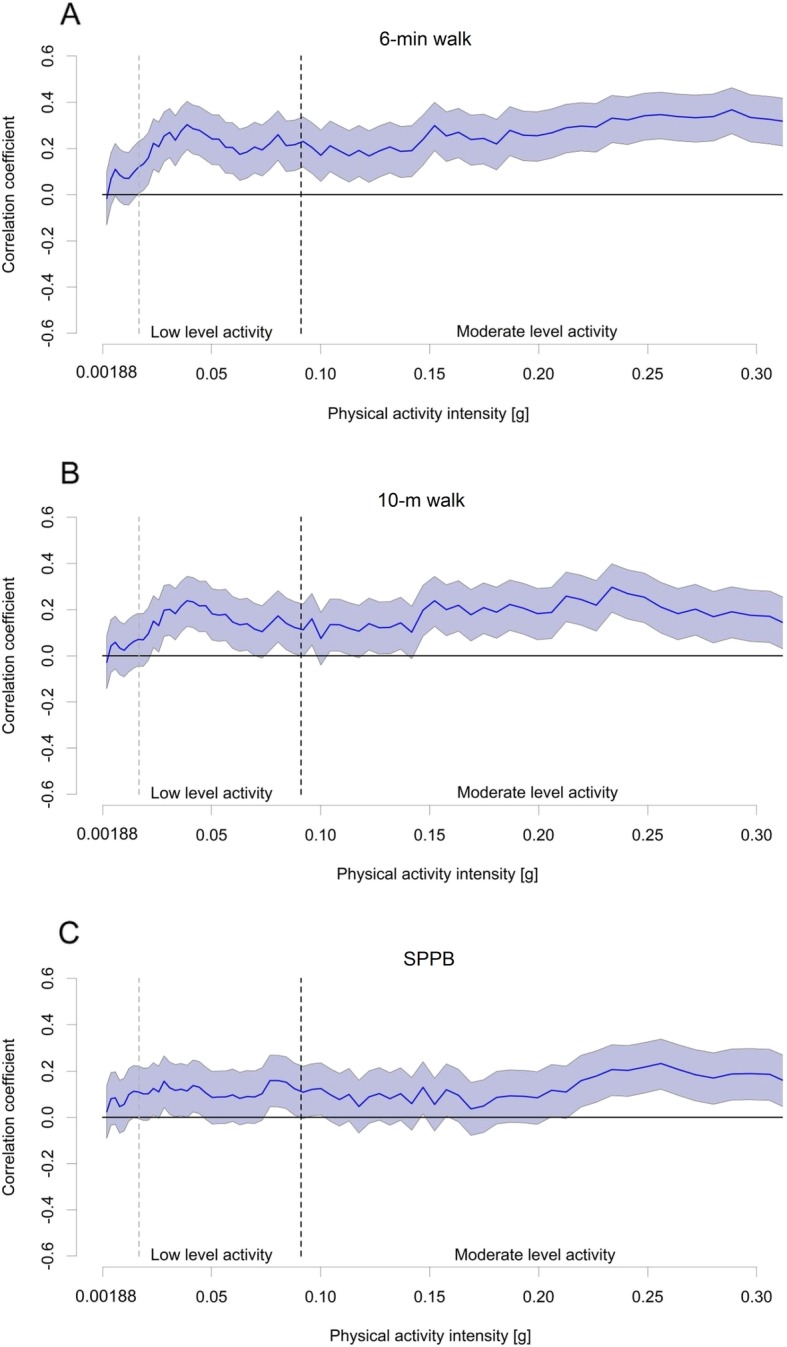
Associations of physical activity intensities from 0.00188 to 0.31 g with physical function. Associations of mean daily minutes at each physical activity intensity bin with 6-min walking distance (**a**), maximal walking speed uver 10 m (**b**) and the SPPB (**c**) are expressed as mean correlation coefficient r (y-axis, black line) and 95% confidence interval (CI, shaded area). Physical activity intensities are shown in the x-axis. Associations are statistically significant, if the 95% CI area does not cross the 0-line. Verticals mark the cut-points for light-intensity activity (0.0167 g) and moderate-intensity activity (0.091 g). Correlations are adjusted by sex and age

When the associations were investigated in detailed intensity ranges, a statistically significant negative association was found between fat percent and mean daily minutes in each of the logarithmically equidistant bins apart from few exceptions, which did not reach statistical significance. Magnitudes of the associations are given in Fig. 3a. For appendicular lean mass, a statistically significant positive association was only found for few narrow intensity ranges at the lower end of moderate-intensity range (Fig. 3b). All activity intensities were positively associated with 6-min walk (Fig. 4a). Associations between 10-m walk and physical activity were statistically significant along almost whole physical activity intensity range (Fig. 4b). SPPB had a significant positive association with physical activity in the higher end of the examined intensity range and in few intensities within the light-intensity range (Fig. 4c).

## Discussion

We found that community-dwelling older adults, who reported to be sedentary or at most moderately physically active, spent most of their waking hours sedentary and in very light-intensity activities. Both light and moderate-to-vigorous activity were associated with lower fat percent and higher walking speed over both long and short distances, and the associations were statistically significant even at very low intensities. In addition, time spent in moderate-to-vigorous-intensity activity had a positive association with lower extremity functioning. More sedentary time was associated with higher fat percent and shorter distance walked in six minutes.

One of our main findings was that light activity was associated with fat percent both as a categorical and as a quasi-continuous measure. Two findings are especially noteworthy. First, the association of light activity with fat percent was almost as strong as that between moderate-to-vigorous activity and fat percent, even after adjusting for moderate-to-vigorous activity. Second, a moderately strong beneficial association was found even for very light-intensity activities. These findings may be explained by that fat percent is sensitive to aerobic activities of any intensity. We measured physical activity in older adults’ normal daily life, and the monitors recorded activity during their daily chores. Light activities common to older adults, such as walking and habitual daily household activities, are well captured with accelerometers [20], and they can contribute substantially to the total energy expenditure [16]. Our findings add to the growing body of evidence, that even lower levels of accelerometer-measured physical activity are negatively associated with obesity among older adults [16].

Interestingly, the association between physical activity and fat percent turned significantly negative already below the cut-point of light-intensity activity, which may have led to underestimation of the association between sedentary time and fat percent. This may indicate that the MAD cut-points defined and validated in healthy younger adults [25, 26] may have been too high in our study population. In a recent study, the optimal MAD cut-point to separate sitting from standing was suggested to be as low as 0.0033 g among children [32]. It may be that the previously defined cut-point of 0.0167 g for light-intensity activity [25] is too high among older adults. A common challenge in measuring physical activity among older adults is that accelerometers do not take into account the age-related decline in physiological functions [21] and the higher energy cost of walking in older age [33]. For example, the intensity of physical activity is often expressed in activity counts, and the most commonly used cut-point for moderate-intensity activity has been shown to underestimate activity intensity among many older adults [22]. Physical activity defined by the standard MAD cut-points may thus be more strenuous for older individuals. Our findings support the previously highlighted need for age-specific or individually tailored cut-points for physical activity intensities [22, 34].

Our finding that physical activity of any intensity was beneficially associated with walking speed over both long and short distance is remarkable, since performance in walking tests predicts disability, mobility limitation and deaths [35]. The association of moderate-to-vigorous-intensity activity with walking speed was expected and in line with previous cross-sectional studies (10–13). In contrast, light-intensity activity has been beneficially associated with walking speed in some [10], but not all [9, 11] studies. One study found a significant association only in women [13]. In the present study, the associations of time spent in light activity with 6-min walk and 10-m walk were statistically significant even after adjusting for time spent in moderate-to-vigorous intensity activity suggesting that light-intensity activity has an independent positive association with walking speed. Another important finding was that even very light-intensity activity was associated with walking speed over both long and short distance. It is worth noting, that the associations of physical activity with walking speed turned positive, even though non-significantly, already below the cut-point for light activity. This may have led to underestimation of the association of sedentary time with walking speed, and can explain why we only found a significant association between sedentary time and 6-min walk whereas other studies have shown a significant association also between sedentary time and other walking tests [10–12]. The disparities may also be due to e.g., differences in study populations, walking tests utilized, physical activity assessment and analysis methods or the statistical analyses performed, which make comparing results from different studies somewhat complicated.

The positive association between accelerometer-based physical activity and walking speed is rational since maximal walking speed and walking endurance are both traits that are sensitive to habitual walking activity, which is common among older adults and well captured with accelerometers [20, 21]. In the present study, physical activity had stronger associations with 6-min walk than with 10-m walk. This may be explained by that 6-min walk represents steady state locomotion, the type of activity best captured with accelerometry, whereas short bursts of high-intensity activity similar to 10-m walk may be dissipated when the activity intensity is averaged into one-minute epochs [25]. Thus, the associations between physical activity and maximal walking speed should be investigated also in shorter epochs in the future.

The association between physical activity and lower extremity functioning assessed with the SPPB test was positive, but more distinct for higher intensities. This is not surprising, since the SPPB is a composite measure and assesses lower extremity strength and balance in addition to habitual walking speed [31]. Activity types that enhance these traits, such as resistance training and yoga, are not well captured with accelerometers [20, 21]. Resistance training is assumed to be more effective for muscle mass than aerobic exercise [6], which may also explain, why we, similar to Westbury et al. [14], did not find any association between physical activity and appendicular lean mass. It may also be that the cross-sectional study setting was not capable to reveal the associations between accelerometer-based physical activity and muscle mass, since Shephard et al. [15] found higher habitual physical activity level to be associated with better maintenance of muscle mass in a longitudinal study. Since accelerometry is limited in assessing the associations of physical activity with lower extremity functioning and muscle mass, utilizing PA diary in addition to accelerometry would be worthwhile, as well as conducting more longitudinal research. Future studies should also take into account participants’ diet and nutrition, since adequate nutrient intake, including e.g., protein and vitamin D, is a key determinant of muscle mass and physical function [36].

We also found that the mean daily time spent within each of the investigated activity intervals declined drastically from very light to moderate-intensity activity, and the amount of vigorous activities was practically non-existing. Less than one third of participants had any activity exceeding 0.31 g, which correspondents to brisk walking (> 5.0 km/h) in a healthy adult population [26]. The mean daily times spent in sedentary and moderate-to-vigorous-intensity activities (10 h and half an hour, respectively) in the present study are in line with findings from recent reviews [19, 34]. This study adds to the literature knowledge about distribution of physical activity throughout the whole intensity range among older adults. Based on the findings from the present study and from the study among children from Gao et al. [32], it is necessary to further investigate especially the lower end of the intensity range and whether the previously defined cut-point to separate sedentary activities from light activities [25] is accurate among older adults.

## Strengths and limitations

The strengths of this study include investigating the distribution of accelerometer-measured physical activity and evaluating the associations of physical activity with body composition and physical function along the whole intensity range. This was a novel analysis approach [21], which provided new information. Another strength is a relatively large, population-based sample of community-dwelling older adults, and assessment of several body composition and physical function variables, which all are meaningful and important for health and physical functioning and thus disability prevention among older adults.

This study also has its limitations. The study design of the XX-study required the participants to be sedentary or at most moderately active, but relatively healthy and community-dwelling, which limits generalizability of our results to all older adults. In agreement with the study design, the amount of higher-intensity activities was low, thus we cannot draw any conclusions on the associations of high-intensity physical activity with body composition and physical function. The activity level of participants was, however, higher than expected. It may be that participants did not consider e.g., walking errands as moderate-intensity activity, when they were initially interviewed for potential exclusion due to meeting the physical activity recommendations, and thus underestimated their physical activity level. According to the previous physical activity recommendations, participants self-reported at least moderate intensity activity bouts lasting at least 10 min. The accelerometer recordings, however, include moderate-to-vigorous intensity activity in bouts of any length, which may have led to higher amounts of moderate-to-vigorous activity than if it would have been investigated only in longer bouts. Third, it may be that participants were excited about the accelerometer measurements and increased their physical activity level during the measurement period. Future research is needed both among physically more active older adults as well as among more sedentary and less healthy and functioning older adults. On the other hand, exploration of this at most moderately active population did lend credence to the emergence that even small increments of light physical activity may confer health benefits to older adults [16].

Due to the cross-sectional nature of this study, any conclusions of causal relationships between physical activity and outcomes cannot be drawn. It may be, that favorable body composition and better physical function lead to higher levels of physical activity, and not vice versa. More longitudinal and experimental research is needed. Accelerometry has also some limitations, as noted previously. The analysis algorithm may neither have been sensitive enough to separate non-wear time from sedentary activities. In some cases, self-reported wear-time was excluded as non-wear time and self-reported non-wear time included as wear-time by the analysis algorithm. Investigating physical activity in detailed intensity ranges utilizing MADs is a novel analysis approach among older adults, and more research utilizing this analysis approach is required to verify the accuracy and applicability of the method.

## Conclusion

In conclusion, the present study expands the understanding of amount and intensity of physical activity and the associations of physical activity with body composition and physical function along the whole intensity range among sedentary or at most moderately active older adults. We found that physical activity of any intensity was beneficially associated with fat percent and walking speed over both long and short distances. These findings provide additional evidence of the importance of encouraging older adults to engage in physical activity of any intensity. It may be that emphasizing moderate-to-vigorous-intensity activity is not feasible, since the majority of this population is unable to engage in high-intensity activities. Conclusive evidence shows, however, that physical activity of at least moderate intensity has a wide range of health benefits [4] and is required for preserving or improving cognitive functioning in older age [37]. To promote adaptation to physically active lifestyle, physical activity counseling among previously sedentary or at most moderately active older populations should thus initially highlight the benefits of increasing the amount of daily light-to-moderate-intensity activity. To gain greater benefits for health and functioning, older adults should be encouraged to increase the intensity of their habitual physical activity gradually. The relationships of light-intensity physical activity with body composition and physical function should be verified in future studies utilizing randomized controlled trial setting.

## Data Availability

The datasets used during the current study are available from the corresponding author on reasonable request.
